# NF-kappaB-dependent MicroRNA-425 upregulation promotes gastric cancer cell growth by targeting PTEN upon IL-1β induction

**DOI:** 10.1186/1476-4598-13-40

**Published:** 2014-02-26

**Authors:** Jun Ma, Jun Liu, Zhiming Wang, Xixi Gu, Yue Fan, Wen Zhang, Lili Xu, Jianjun Zhang, Dingfang Cai

**Affiliations:** 1 Department of Integrative Medicine, Zhongshan Hospital, Fudan University, Shanghai, PR China; 2 Department of Oral & Maxillofacial-Head Neck Oncology, Ninth People’s Hospital, Shanghai Jiao Tong University School of Medicine, Shanghai, PR China; 3 Department of Oncology, Zhongshan Hospital, Fudan University, Shanghai, PR China

**Keywords:** IL-1β, NF-kappaB, miR-425, PTEN, Gastric cancer

## Abstract

Overexpression of the proinflammatory cytokine IL-1β is associated with diverse diseases, including cancer. Alteration of microRNAs has been observed in cancer cells exposed to proinflammatory cytokines, yet their function in inflammation stress remains elusive. Here, we show that IL-1β induces the upregulation of miR-425, which negatively regulates phosphatase and tensin homolog expression by targeting its 3’ UTR. An increase in miR-425 depends on IL-1β-induced NF-kappaB activation, which enhances miR-425 gene transcription upon IL-1β induction. Consequently, repression of phosphatase and tensin homolog by miR-425 promotes gastric cancer cell proliferation, which is required to protect cells from cisplatin-induced apoptosis. Taken together, our data support a critical role for NF-kappaB-dependent upregulation of miR-425, which represents a new pathway for the repression of phosphatase and tensin homolog activation and the promotion of cell survival upon IL-1β induction.

## Background

Gastric adenocarcinoma is the fourth and fifth most common cancer among males and females, respectively, worldwide and is strongly linked to chronic inflammation [1]. It is now well accepted that infection with *Helicobacter pylori* (*H. pylori*) plays a major role in triggering chronic inflammation leading to malignancy [2]. Chronic inflammation of the stomach initiates the histopathological progression of chronic gastritis to gastric atrophy, intestinal metaplasia and finally gastric cancer [3]. While *H. pylori* infection is extremely prevalent, only a small minority (approximately 1%) of infected individuals will develop gastric cancer after many years. The variable response to this common pathogen appears to be governed by a genetic predisposition to high expression levels of proinflammatory cytokines [4].

The nuclear factor kappa B (NF-kappaB) pathway has long been considered a major proinflammatory signaling pathway, largely based on the activation of NF-kappaB by proinflammatory cytokines and the role of NF-kappaB in the transcriptional activation of responsive genes including cytokines and chemokines [5]. The “canonical” pathway for NF-kappaB activation is triggered by proinflammatory cytokines such as IL-1β and usually leads to the activation of RelA- or cRel-containing complexes [6]. NF-kappaB exists in the cytoplasm in an inactive form associated with regulatory proteins referred to as inhibitors of κB (IκB), of which the most important may be IκBα, IκBβ, and IκBϵ. IκBα is associated with transient NF-kappaB activation, whereas IκBβ is involved in sustained activation [7]. However, chronic inflammation is a complex physiological process, and the role of NF-kappaB in the inflammatory response has not yet been fully explored.

In addition to affecting protein-coding gene expression, inflammation stress also changes the expression level of microRNAs (miRNAs) [8]. MicroRNAs are a class of endogenous, small, non-coding RNAs that negatively regulate gene expression at the post-transcriptional level mainly via binding to the 3’ untranslated region of a target mRNA, and they have important regulatory functions in the control of diverse physiological and pathological processes [9,10]. These RNAs have been shown to be involved in the regulation of many cellular processes including proliferation, differentiation, and apoptosis [11-13]. However, whether chronic inflammation regulates miRNA expression by modulating gene transcription or altering post-transcriptional maturation has not been determined.

In this work, we found that miR-425 induction upon IL-1β-induced inflammation was dependent on the activation of NF-kappaB, which enhanced miR-425 gene transcription. Moreover, the upregulated miR-425 directly targeted phosphatase and tensin homolog (PTEN) and negatively regulated its expression, which promoted cell survival upon IL-1β induction.

### Experimental procedures

#### Ethics statement

All specimens were obtained from patients who underwent surgery at Fudan University Shanghai Cancer Center. The protocol was approved by the Clinical Research Ethics Committee of Fudan University, and the research was carried out according to the provisions of the Helsinki Declaration of 1975. Adjacent normal tissues were excised away from the gastric cancer lesion macroscopically, and their histological diagnosis was confirmed microscopically. Written informed consent was obtained from all participants involved in the study.

### Cell culture and reagents

The human embryonic kidney cell line HEK293 (ATCC® CRL-1573™), the human breast cancer cell line MDA-MB361 (ATCC® HTB-27™), the human gastric adenocarcinoma cell line AGS (ATCC® CRL-1739™), SNU-1 (ATCC® CRL-5971™), SNU-5 (ATCC® CRL-5973™), SNU-16 (ATCC® CRL- 5974™), Hs746T (ATCC® HTB-135™), NCI-N87 (ATCC® CRL-5822™), and KATO III (ATCC®HTB-103™) were maintained in DMEM containing 10% fetal bovine serum. All cell lines were maintained in media containing penicillin (100 IU/ml) and streptomycin (100 mg/ml) at 37°C with 5% CO_2_. The miRNA mimics and anti-miRNA were purchased from Ambion (Austin, TX, USA). The IKK inhibitor TPCA-1 (Cat. No. S2824), the p38 MAPK inhibitor BIX02188 (Cat. No. S1574) and the JNK inhibitor SP600125 (Cat. No. S1460) were purchased from Selleckchem (Houston, TX, USA). Recombinant human IL-1β were purchased from Sigma-Aldrich (Cat. No. H6291, Shanghai, China).

### RNA extraction and real-time PCR

Total RNA was extracted from cells using TRIzol (Invitrogen, Carlsbad, CA). For microRNA analysis, poly(A) tails were added to total RNA using poly(A) polymerase (Ambion, Carlsbad, CA) prior to reverse transcription. The MiRcute miRNA qPCR detection kit (TIANGEN, Beijing, China) was used to quantitate the expression levels of mature miR-425 according to the provided protocol, and GAPDH was used as an internal control. Real-time PCR was performed under the following conditions: 95°C 10 m, 1 cycle; 95°C 10 s, 55°C 34 s, 40 cycles.

For all results obtained by real-time PCR methods, we used the delta delta CT method to calculate the fold change in gene expression between different groups. The amount of target (PTEN/miR-425), normalised to the endogenous housekeeping gene GAPDH and relative to a reference sample, is given by the following equation: amount of target =2^-△△CT^.

### Immunoblotting

Proteins were separated on a 10% SDS-PAGE gel and subsequently transferred to a PVDF membrane. After blocking with 5% nonfat milk, the membrane was incubated with a mouse monoclonal anti-PTEN antibody (1:500, Santa Cruz, sc-7974) and a NF-kappaB p65 Phospho (pS536) (RELA) antibody (1:10000, EPITOMICS, Cat.#: 2220–1). IRdye-labeled secondary antibodies were used for quantitation of the immunoblotting signal, and the signals were analyzed using an Odyssey scanner (LI-COR Biosciences, Lincoln, NE, USA).

### Luciferase assay

HEK293 cells and AGS cells were transfected with miR-425 and pGL3 luciferase reporter constructs harboring the miR-425 target sequence. After 24 h, the activities of firefly luciferase and renilla luciferase in the cell lysates were measured with the Dual-Luciferase Assay System (Promega, Madison, WI, USA). For the luciferase transcription reporter assay, miR-425 gene promoter sequences (WT or site deletion) were cloned into the promoter region of the pGL3-Basic vector, and luciferase activity was measured as described above.

### Chromatin immunoprecipitation (ChIP)

Briefly, treated cells were cross-linked with 1% formaldehyde, sheared to an average size of 400 bp, and subsequently immunoprecipitated with antibodies against NF-kappaB (Santa Cruz, sc-166588). The ChIP-PCR primers were designed to amplify the promoter regions containing putative NF-kappaB binding sites within miR-425 as illustrated. A positive control antibody (RNA polymerase II) and a negative control non-immune IgG were used to demonstrate the efficacy of the kit reagents (Epigentek Group Inc, P-2025-48). Immunoprecipitated DNA is then cleaned, released, and eluted. Eluted DNA can be used for downstream applications ChIP-PCR. Fold enrichment (FE) was calculated by using a ratio of amplification efficiency of the ChIP sample over that of non-immune IgG. Amplification efficiency of Polymerase RNA II was used as a positive control. FE% = 2^(IgG CT – Sample CT)^ × 100%.

### Cell proliferation assay

A cell proliferation assay was performed using the Cell Counting Kit-8 (Dojindo, Kumamoto, Japan) according to the manufacturer's instructions. Before the addition of CCK-8, the cells were washed with warm culture media by spinning the plate at 500 rpm for 3 m and then discarding the supernatant.

### Cell apoptosis assay

The cancer cells were harvested and resuspended in 500 μl of a binding buffer. The cell suspension (100 μl) was incubated with 5 μl annexin-V and propidium iodide at room temperature for 20 minutes. The stained cells were analyzed with fluorescent-activated cell sorting (FACS) using BD LSR II flow cytometry.

### Cell cycle analysis

For the flow cytometry analysis, cells were trypsinized and fixed in 70% ethanol overnight. The cells were then incubated in 0.5 ml of propidium iodide solution containing 25 μg ml^−1^ RNase for 15 minutes at 37°C and measured.

### Mouse experiments

The NCI-N87 cells (3 × 10^6^) were injected into the right flanks of athymic nu/nu mice. One week after the injections, mice with comparably sized tumors were treated for 4 weeks with anti-miR-425. The anti-miR-425 (2 nmol) were injected directly into the tumors twice weekly for 4 weeks.

### Statistical analysis

The results are presented as means ± SEM, and the data were analyzed with Student’s t test. A value of *p* < 0.05 was considered statistically significant.

## Results

### IL-1β treatment induces miR-425 expression

**Figure 1:**
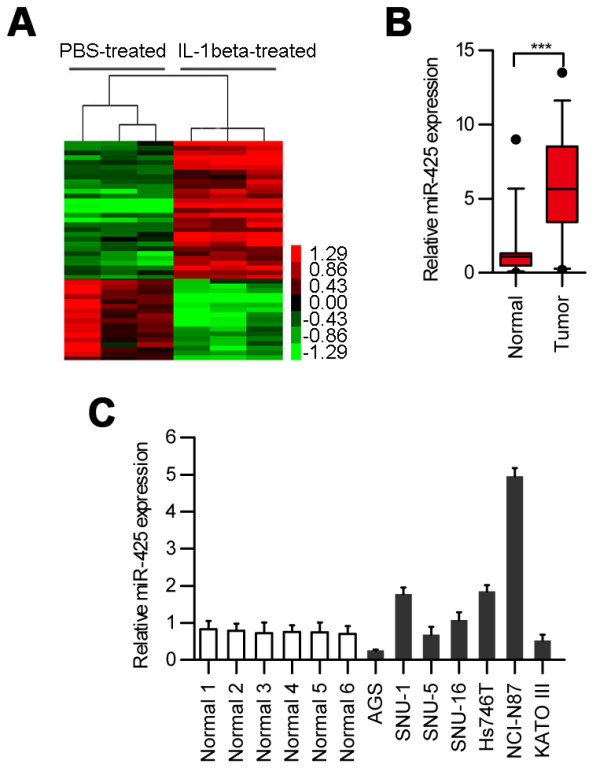
**Dysregulation of miRNAs in human AGS cells treated with IL-1β. (A)** Human AGS cells were treated with IL-1β (10 ng/ml) [14], and 24 h later, the miRNAs expression profile was analysed with microarray technology. Heat map diagram generated by unsupervised clustering analysis with 46 significantly dysregulated miRNAs. Red indicates upregulation; green indicates downregulation. **(B)** Increased levels of miR-425 in 36 tumor samples relative to their levels in matched adjacent normal tissues as measured by real-time PCR. Normal: adjacent normal tissues. **(C)** Expression level of miR-425 was examined by real-time PCR in multiple gastric cancer cell lines and six normal gastric mucosa cells.

To characterize the miRNAs responsible for IL-1β induction, we profiled miRNA expression in PBS-treated AGS cells and IL-1β-induced AGS cells using the Exiqon miRCURY™ LNA Array System (v.14.0). The miRNA levels differed greatly between the PBS-treated group and the IL-1β-induced group, as illustrated in the heat map shown in Figure 1A. Among the 1,891 capture probes, 46 miRNAs were differentially expressed in IL-1β-induced AGS cells compared with paired PBS-treated AGS cells; of these miRNAs, 29 were increased and 17 were decreased in the IL-1β-induced AGS cells (Table 1), indicating that a specific miRNA pattern is associated with IL-1β induction.

**Table 1:** Significant dysregulation of miRNAs

**Overexpressed in IL-1β induced-AGS cells**		
*** miRNA***	***Fold change***	***p value***
**hsa-miR-425**	**12.36**	**0.00**
hsa-miR-584	11.03	0.01
hsa-miR-31	8.12	0.00
hsa-miR-155	6.22	0.00
hsa-let-7i	5.55	0.00
hsa-miR-21	5.11	0.00
hsa-miR-335	4.89	0.01
hsa-miR-191	4.73	0.03
hsa-miR-519d	4.37	0.02
hsa-miR-520d-5p	3,95	0.00
hsa-miR-331	3.67	0.01
hsa-miR-142	3.45	0.01
hsa-miR-518a-5p	3.28	0.01
hsa-miRPlus-F1231	3.11	0.01
hsa-miR-2113	2.94	0.02
hsa-miR-196a*	2.88	0.02
hsa-miR-1304	2.78	0.02
hsa-miR-185	2.65	0.01
hsa-let-7a	2.61	0.00
hsa-miR-130	2.52	0.02
hsa-miR-1280	2.50	0.01
hsa-miR-222	2.47	0.01
hsa-let-7c	2.43	0.00
hsa-miR-602	2.31	0.00
hsa-miR-548e	2.31	0.03
hsa-miR-32	2.27	0.04
hsa-miR-181a	2.24	0.02
hsa-miR-7	2.13	0.00
hsa-miR-21*	2.06	0.00
**Underexpressed in IL-1β induced-AGS cells**		
*** miRNA***	***Fold change***	***p value***
hsa-miR-143	0.06	0.02
hsa-miR-200	0.08	0.01
hsa-miR-451	0.13	0.01
hsa-miR-144	0.17	0.00
hsa-miR-363	0.20	0.01
hsa-miR-637	0.25	0.03
hsa-miR-153	0.39	0.01
hsa-miR-139	0.39	0.00
hsa-miR-617	0.42	0.01
hsa-miR-1915	0.43	0.00
hsa-miRPlus-F1153	0.45	0.00
hsa-miR-381	0.46	0.00
hsa-miR-145	0.47	0.02
hsa-miR-659	0.47	0.03
hsa-miR-125b	0.48	0.00
hsa-miR-183*	0.49	0.00
hsa-miR-638	0.49	0.01

*:When two mature miRNAs originate from opposite arms of the same pre-miRNA, an asterisk following the name indicates an miRNA expressed at low levels relative to the miRNA in the opposite arm of a hairpin.

Among these miRNAs, miR-425 was the most highly upregulated upon IL-1β induction. Using real-time PCR analysis, we analyzed miR-425 expression in 36 paired samples (tumor and adjacent normal tissues from the same patient). We found a significantly higher level of miR-425 expression in the tumor samples relative to the levels in the adjacent normal tissues (*p* < 0.0001) (Figure 1B). We examined the expression level of miR-425 in a set of gastric cancer cell lines and six normal gastric mucosa cells. As shown in Figure 1C, we picked up the AGS cells with down-regulated miR-425 and the NCI-N87 cells with up-regulated miR-425 for further study. Although the activation of miR-425 has been reported to have a fundamental impact on cancer initiation and progression of cancer cells by reducing the expression of an extensive network of genes [15], the role of miR-425 in human cancers has not been elucidated. We therefore chose miR-425 for further investigation.

### Expression of PTEN is negatively regulated by miR-425

**Figure 2:**
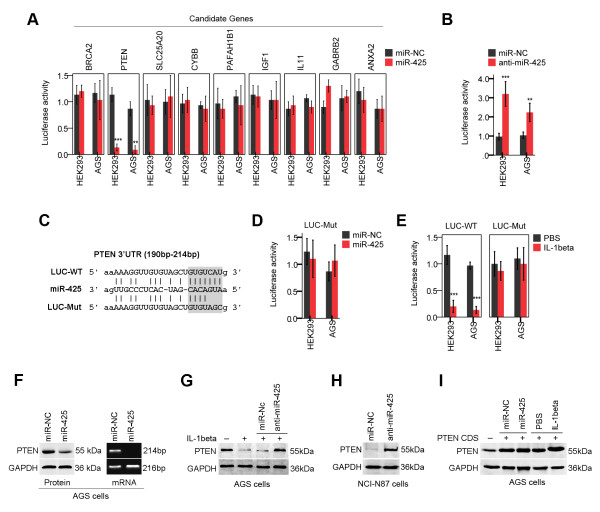
**miR-425 directly targets PTEN. (A)** Reporter assay in HEK293 cells and AGS cells transfected with miR-425 and constructs carrying luciferase cDNA fused to the 3’ UTRs of predicted candidate targets (mean ± SEM). **(B)** Effects of anti-miR-425 on the luciferase activity of luciferase constructs fused with the 3’ UTRs of PTEN in HEK293 cells and NCI-N87 cells (mean ± SEM). **(C, D)** Reporter assay in HEK293 cells and AGS cells transfected with luciferase constructs carrying PTEN 3’ UTRs with mutations in the miR-425-binding sites (mean ± SEM). **(E)** HEK293 cells and AGS cells were transfected with a luciferase construct carrying the wild type PTEN 3’ UTR (LUC-WT) or a construct carrying a mutated PTEN 3’ UTR (LUC-Mut). After 24 h, the cells were treated with IL-1β, and luciferase activity was quantified 24 h after treatment. **(F)** Western blot and RT-PCR showing PTEN protein levels and mRNA levels in AGS cells after 48 h of miR-425 transfection. **(G)** AGS cells were transfected with anti-miR-425, and 24 h later, the cells were either left untreated or treated with IL-1β and subsequently harvested 24 h after treatment. Whole cell lysates were immunoblotted with PTEN antibodies. **(H)** NCI-N87 cells were transfected with anti-miR-425 and harvested 24 h after treatment. Whole cell lysates were immunoblotted with PTEN antibodies. **(I)** AGS cells were transfected with a plasmid carrying only the open reading frame sequence of PTEN (PTEN-CDS). After 24 h, the cells were treated with IL-1β or miR-425. Whole cell lysates were subjected to immunoblotting after 24 h of treatment. (*p < 0.05; **p < 0.01; ***p < 0.001).

To identify the targets of miR-425, we employed a commonly used algorithm, miRecords (http://mirecords.biolead.org/), which is an integrated resource for animal miRNA-target interactions. To increase the accuracy of this prediction, genes that were predicted by at least five of eleven databases (Diana, microinspector, miranda, mirtarget2, mitarget, nbmirtar, pictar, pita, rna22, rnahybrid and targetscan) were selected as putative targets. Among these putative targets of miR-425, gene ontology analysis revealed that the expression levels of 9 candidate genes were altered; thus, this alteration could contribute to the malignant phenotype. Using 3’ UTR luciferase reporter assays, we found that overexpression of miR-425 significantly inhibited luciferase activity in HEK293 cells and AGS cells expressing the wild type PTEN 3’ UTR reporter (Figure 2A). We confirmed that PTEN is a putative direct target of miR-425. To illustrate the specificity of miR-425, we showed that anti-miR-425 specifically abolished the inhibition of luciferase activity induced by miR-425 in HEK293 cells and NCI-N87 cells (Figure 2B). Mutations in the miRNA binding sites (Figure 2C) rendered the constructs unresponsive to miR-425 induction (Figure 2D), further confirming that the PTEN gene is a direct target of miR-425.

Furthermore, mutation of the miR-425 target sequence also significantly attenuated IL-1β-induced repression of PTEN 3’ UTR luciferase reporter activity in HEK293 cells and AGS cells (Figure 2E). Overexpression of miR-425 was sufficient to downregulate PTEN expression at both the protein and mRNA levels in AGS cells (Figure 2F). Accordingly, IL-1β-induced PTEN repression was rescued by expressing anti-miR-425 in AGS cells (Figure 2G). Anti-miR-425 was able to up-regulate PTEN expression in NCI-N87 cells without IL-1β stimulation (Figure 2H).

Our data also indicated that the 3’ UTR is required for miR-425-mediated PTEN downregulation because expression of a PTEN coding region construct (PTEN-CDS) was insensitive to miR-425 overexpression and IL-1β induction in AGS cells (Figure 2I). Taken together, these results indicate that miR-425 plays a critical role in repressing PTEN expression by targeting its 3’ UTR upon IL-1β induction.

### IL-1β-induced NF-kappaB activation is required for miR-425 induction

**Figure 3:**
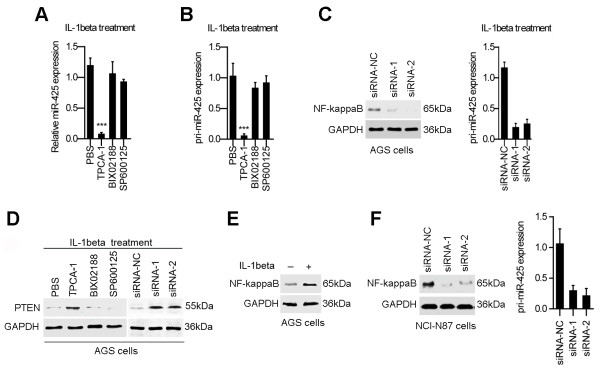
**IKK activation is required for the induction of miR-425 in response to IL-1β induction. (A)** AGS cells were treated with IL-1β in the presence of the IKK inhibitor TPCA-1, the p38 MAPK inhibitor BIX02188 and the JNK inhibitor SP600125. Mature miR-425 expression at 12 h after treatment was quantitated by real-time PCR. The fold change of relative miR-425 expression (miR-425/U6) was normalized to that observed in PBS-treated cells. **(B)** The expression of primary miR-425 (pri-miR-425) was analyzed by real-time PCR as in A. **(C)** The expression levels of NF-kappaB protein and pri-miR-425 were analyzed by real-time PCR in AGS cells treated with siRNAs for NF-kappaB. **(D)** AGS cells were treated with chemistry inhibitors or siRNAs for NF-kappaB. Cell lysates were obtained 24 h after IL-1β treatment and immunoblotted with PTEN antibodies. **(E)** Western blot analysis of phosphorylated NF-kappaB p65 (serine 536). **(F)** The expression levels of NF-kappaB protein and pri-miR-425 were analyzed by real-time PCR in NCI-N87 cells treated with siRNAs for NF-kappaB. (*p < 0.05; **p < 0.01; ***p < 0.001).

To determine the mechanism involved in miR-425 transactivation upon IL-1β induction, we examined the impact of various kinase inhibitors on miR-425 induction in IL-1β-treated AGS cells. IL-1β-induced miR-425 upregulation was significantly inhibited by the IKK inhibitor TPCA-1 but not by the p38 MAPK inhibitor BIX02188 or the JNK inhibitor SP600125 (Figure 3A). Previous studies have demonstrated that IKK is an essential kinase required for NF-kappaB signaling; therefore, this result indicated the critical role of NF-kappaB signaling in the regulation of miR-425 transcription upon IL-1β induction.

Consistently, induction of pri-miR-425 upon IL-1β treatment was remarkably inhibited in the presence of the IKK inhibitor or siRNAs for NF-kappaB (Figure 3B and C). We also observed that IL-1β-induced PTEN repression was attenuated in the presence of the IKK inhibitor or siRNAs for NF-kappaB (Figure 3D). To determine whether NF-kappaB activity was present in AGS cells treated with IL-1β, we used a western blot to determine the level of phosphorylated NF-kappaB p65 (serine 536). The level of phosphorylated NF-kappaB p65 (serine 536) was high in AGS cells treated with IL-1β (10 ng/ml; 24 hours) (Figure 3E). In addition, silencing of NF-kappaB inhibited miR-425 expression in NCI-N87 cells without IL-1β treatment (Figure 3F). These results suggesting that IKK-dependent NF-kappaB activation upon IL-1β treatment is required for PTEN downregulation, most likely via its enhancement of miR-425 transcription.

**Figure 4:**
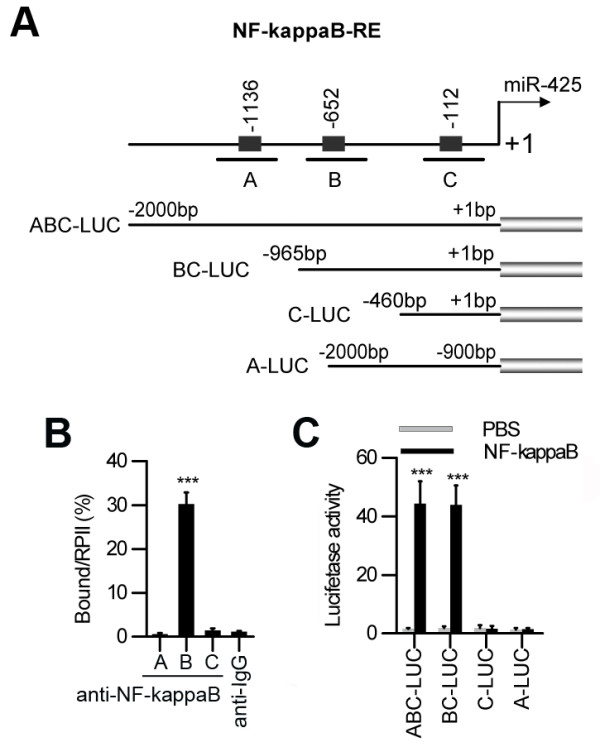
**NF-kappaB binding sites in the miR-425 promoter. (A)** Features of the miR-425 5’ flanking DNA. Human promoter regions of miR-425 contain three putative binding sites for NF-kappaB. **(B)** ChIP assays with anti-NF-kappaB antibodies showed binding of NF-kappaB to the promoter of miR-425 in AGS cells. The relative occupancies of NF-kappaB are indicated as vertical bars. The bar graphs show the averages of three independent ChIP experiments. **(C)** The promoter of miR-425 was activated by NF-kappaB. AGS cells were treated with NF-kappaB and transfected with the indicated LUC vectors.

To determine whether NF-kappaB directly regulates miR-425 transcription, we analyzed the upstream sequences of miR-425 using the WeightMatrix library (matrixTFP60.lib) and identified three potential NF-kappaB binding sites in the promoter region of miR-425 (cut-offs: matrix similarity = 0.9 and core similarity = 0.95) (Figure 4A). We performed chromatin immunoprecipitation (ChIP) assays with AGS cancer cells using monoclonal anti-NF-kappaB antibodies. As shown in Figure 4B, only primer-B of miR-425 produced strong PCR products, which suggested that the NF-kappaB protein formed complexes with the B binding site in the miR-425 promoter. The results of luciferase reporter assays suggested that the potential B binding site in the miR-425 promoter is required for transactivation of the downstream gene upon IL-1β induction (Figure 4A and C).

### Induction of miR-425 promotes cell survival upon IL-1β induction

**Figure 5:**
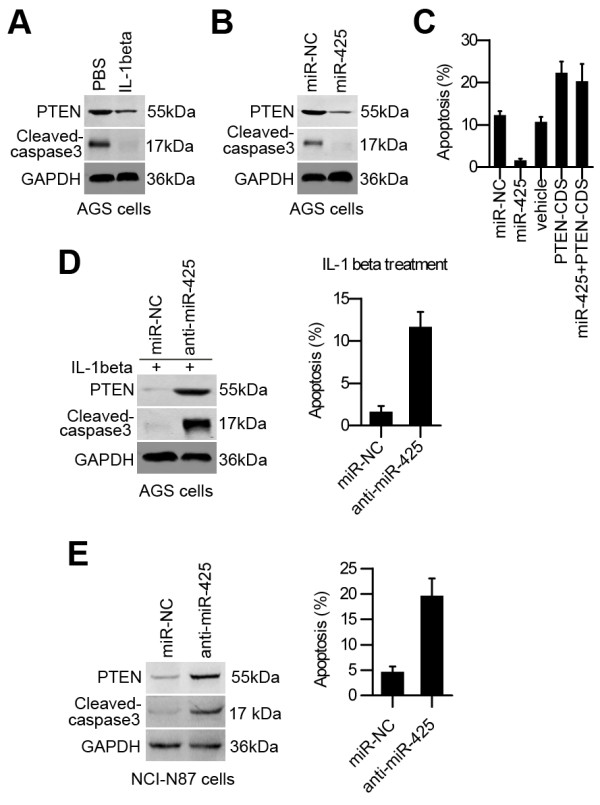
**miR-425 inhibits cell apoptosis by repressing PTEN. (A)**. AGS cells were treated with control (PBS) or IL-1β. After 48 h, whole cell lysates were immunoblotted. **(B)**. AGS cells were transiently transfected with negative control (miR-NC) or miR-425 alone. After 24 h, the cells were treated with cisplatin and harvested 24 h after treatment. Whole cell lysates were immunoblotted. **(C)**. AGS cells were transfected with miR-NC, miR-425, and PTEN-CDS as indicated. After 24 h, the cells were treated with cisplatin and harvested 24 h after treatment. The cell apoptosis rate was determined using the flow cytometry method. **(D)**. AGS cells were transfected with miR-NC or anti-miR-425. After 48 h, the cells were treated with IL-1β and harvested 24 h after treatment. Total cell lysates were immunoblotted with the indicated antibodies, and the cell apoptosis rate was determined using the flow cytometry method. **(E)** NCI-N87 cells were transfected with miR-NC or anti-miR-425. Total cell lysates were immunoblotted with the indicated antibodies, and the cell apoptosis rate was determined using the flow cytometry method.

It was shown that PTEN is among the most frequently inactivated tumor suppressor genes. Overexpression of PTEN in different mammalian tissue culture cells affects various processes including cell proliferation, cell death and cell migration [16]. We also found that inhibiting PTEN decreased the activation of caspase-3 in cells treated with IL-1β (Figure 5A). It is plausible that miR-425 induction may inhibit apoptosis via the downregulation of PTEN in IL-1β-treated cells. Indeed, overexpression of miR-425 inhibited caspase-3 activation in cisplatin-treated AGS cells (Figure 5B). Moreover, in cisplatin-treated AGS cells, cotransfection of a construct containing only the PTEN coding region (PTEN-CDS), which is insensitive to miR-425, bypassed the antiapoptotic effect of miR-425 overexpression (Figure 5C). Accordingly, transfection of anti-miR-425 in AGS cells significantly enhanced caspase-3 activation and apoptosis in response to IL-1β treatment (Figure 5D). In addition, transfection of anti-miR-425 in NCI-N87 cells significantly enhanced caspase-3 activation and apoptosis without IL-1β stimulation (Figure 5E).

**Figure 6:**
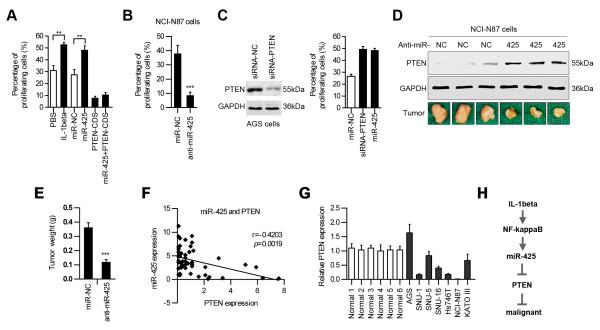
**miR-425 promotes cell proliferation by repressing PTEN. (A)** AGS cells were treated with PBS, IL-1β, miR-NC, miR-425, and PTEN-CDS as indicated. After 72 h, cell proliferation was determined using the EdU labeling kit. **(B)** NCI-N87 cells were transfected with miR-NC or anti-miR-425. After 72 h, the cell proliferation rate was determined using the EdU labeling kit. **(C)** AGS cells were treated with siRNA-PTEN or miR-425. After 72 h, cell proliferation was determined using the EdU labeling kit. **(D)** Photographs of PTEN protein expression in the tumor tissues (upper panels) and tumors (lower panels). **(E)** The graph shows the weight of the 3 tumors after 4 weeks (n = 3). **(F)** A significant inverse correlation is observed between the miR-425 and PTEN expression levels in the gastric cancer tissues (n = 52). **(G)** The expression levels of PTEN are determined in six normal gastric mucosa cells and gastric cancer cell lines using real-time PCR. **(H)** A model illustrating the putative roles of IL-1β and miR-425 in the control of the PTEN pathway in human gastric carcinoma cells.

Consistent with its role in inhibiting caspase activation, upregulation of miR-425 substantially enhanced AGS cell proliferation, whereas the pro-survival effect was completely blocked by co-transfection with exogenous PTEN (PTEN-CDS) (Figure 6A). Anti-miR-425 decreased the percentage of proliferating cells for NCI-N87 cells (Figure 6B). We also found that inhibiting PTEN had a protective effect similar to that observed in cells overexpressing miR-425 (Figure 6C), suggesting that PTEN repression may play a major role in miR-425-dependent protection in cells treated with IL-1β.

We investigated the effect of miR-425 on tumorigenicity in vivo. The tumors treated with anti-miR-425 showed increased levels of the PTEN protein (Figure 6D). Also, anti-miR-425 reduced the tumor weight of the mice compared with the miR-NC-treated group (Figure 6E). Using non-parametric tests, we found a significant inverse correlation between PTEN mRNA and miR-425 expression in the gastric cancer samples (Figure 6F). The expression levels of PTEN were also determined in six normal gastric mucosa cells and gastric cancer cell lines using real-time PCR. As shown, the cells with “down-regulated” miR-425 have higher amounts of PTEN compared to cell lines with “up-regulated” miR-425 levels (Figure 6G). In conclusion, our results have proven that miR-425 plays a causal role through targeting PTEN in gastric cancers.

## Discussion

Interleukin-1 (IL-1) is a major pro-inflammatory cytokine that is produced by malignant or microenvironmental cells [17]. IL-1 also functions as a pleiotropic cytokine involved in tumorigenesis and tumor invasiveness; therefore, it represents a feasible candidate for a modulatory cytokine that can tilt the balance between inflammation and immunity toward the induction of antitumor responses [18]. IL-1α and IL-1β are the major agonists of IL-1. In their secreted forms, IL-1α and IL-1β bind to the same receptors and induce the same biological functions [19]. However, IL-1α and IL-1β differ in their compartmentalization within the cell or the microenvironment [20]. IL-1β is only active in its secreted form and mediates inflammation, which promotes carcinogenesis, tumor invasiveness and immunosuppression [21]. Some novel anti-IL-1β agents have been used in clinical trials in patients exhibiting diverse diseases with inflammatory manifestations [22]. A better understanding of the integrative role of IL-1β signaling pathways in the malignant process will enable the application of novel IL-1β modulation approaches in cancer patients.

PTEN was discovered as an important tumor suppressor that is often mutated or lost in various cancers [23]. Several lines of evidence have highlighted PTEN as a lipid phosphatase that hydrolyzes the 3’ phosphate in phosphoinositides [24]. PTEN can also regulate the activity of the serine/threonine kinase AKT/PKB and can thus influence cell survival signaling [25]. UV exposure can trigger PTEN interaction with wild-type melanocortin-1 receptor variants, which protects PTEN from WWP2-mediated degradation, leading to AKT inactivation in melanoma [26]. There are multiple mechanisms for the regulation of PTEN, including transcription, mRNA stability, microRNA targeting, translation and protein stability. PTEN is transcriptionally silenced by promoter methylation in gastric carcinoma [27]. PTEN can also be post-translationally regulated by acetylation, ubiquitylation, oxidation, phosphorylation, and subcellular localization [28]. Despite extensive characterization of PTEN mutations in human cancers and a relatively good understanding of the molecular roles of PTEN in the control of cellular processes, little is known about modes of PTEN regulation.

PTEN can be inhibited in cancer cells upon induction of the pro-inflammatory cytokine IL-1β [29]. Stimulation with IL-1β activates NF-kappaB by phosphorylation and degradation of IκB. This activation allows NF-kappaB to translocate into the nucleus and transcriptionally activate target genes [30]. NF-kappaB is a heterodimeric transcription activator consisting of the DNA binding subunit p50 and the transactivation subunit p65 [31]. High levels of endogenous NF-kappaB decreased the expression of PTEN, and PTEN expression could be rescued by specific inhibition of the NF-kappaB pathway [32]. These findings indicate that NF-kappaB activation is necessary and sufficient for the inhibition of PTEN expression. Importantly, the mechanism underlying suppression of PTEN expression by NF-kappaB was independent of p65 transcription function [33]. These studies indicate that other molecules may be involved in the process of PTEN expression inhibition by NF-kappaB.

In this study, we described a novel signaling pathway in which miR-425 can negatively control PTEN activation in cells upon IL-1β induction. The IL-1β-induced expression of miR-425 was regulated by NF-kappaB. Selective inhibition of PTEN by siRNA or miR-425 can improve cell survival in response to IL-1β treatment. However, we cannot rule out the possibility that IL-1β could induce additional miRNAs that could directly or indirectly target PTEN. We presume that there are other IL-1β-induced miRNAs involved in regulating PTEN expression because overexpression of anti-miR-425 could not completely block PTEN repression (Figure 2G). In addition to miR-425, miR-21 [34] and miR-32 [35] have been shown to target PTEN and to modulate growth, migration, and invasion in cancers of the digestive system. Downregulation of PTEN by miR-21 and miR-32 significantly enhanced the survival and proliferation of human cancer cells exposed to inflammation stress, further supporting a critical role for PTEN in the mediation of apoptosis.

NF-kappaB activation is generally considered to be pro-survival. We found that IL-1β-induced NF-kappaB activation was required for the upregulation of miR-425, which promoted cell survival by repressing PTEN. NF-kappaB was also considered as one of the major contributors in the oncogenesis of chronic inflammation-induced colorectal carcinomas, most likely through the upregulation of its pro-survival target genes including cyclin D1, VEGF, IL-8, COX2, and MMP9 [36]. Therefore, the impact of NF-kappaB activation on cell survival and proliferation in response to chronic inflammation most likely needs to be weighed in the context of cell types and cytokines as well as the extent of activation. Similarly, the role of miR-425 in the regulation of cell growth and tumor progression is being studied but remains inconclusive. The oncogenic function of miR-425 was associated with reduced expression of genes such as stab1, ccnd2, and fscn1 [15]. The role of miR-425 in solid tumors is relatively unknown.

Taken together, our data support the critical role of NF-kappaB-dependent upregulation of miR-425, which represents a new pathway for the repression of PTEN activation and the promotion of cell survival upon IL-1β induction. Our studies will aid researchers searching for novel putative therapeutic markers.

## Competing interest

The authors declare no competing financial interests.

## Authors' contribution

JM carried out the molecular biology studies. JL drafted the manuscript. ZW carried out the bioinformatic analysis. XG and YF participated in the cell migration and invasion assays. WZ carried out the immunoblotting analysis. LX performed the statistical analysis. JZ and DC participated in the design of the study and coordination and helped to draft the manuscript. All authors read and approved the final manuscript.
